# Glial Purinergic Signaling-Mediated Oxidative Stress (GPOS) in Neuropsychiatric Disorders

**DOI:** 10.1155/2022/1075440

**Published:** 2022-03-04

**Authors:** Lumei Huang, Yong Tang, Beata Sperlagh

**Affiliations:** ^1^Laboratory of Molecular Pharmacology, Institute of Experimental Medicine, Budapest, Hungary; ^2^János Szentágothai Doctoral School, Semmelweis University, Budapest, Hungary; ^3^International Collaborative Centre on Big Science Plan for Purinergic Signalling, Chengdu University of Traditional Chinese Medicine, Acupuncture and Chronobiology Key Laboratory of Sichuan Province, Chengdu, China

## Abstract

Oxidative stress (OS) has been implicated in the progression of multiple neuropsychiatric disorders, including schizophrenia (SZ), major depressive disorder (MDD), bipolar disorder, and autism. However, whether glial purinergic signaling interaction with oxidative/antioxidative system displays an important role in neuropsychiatric disorders is still unclear. In this review, we firstly summarize the oxidative/antioxidative pathways shared in different glial cells and highlight the cell type-specific difference in response to OS. Then, we collect the evidence showing the regulation of purinergic signaling in OS with an emphasis on adenosine and its receptors, P2Y1 receptor in the P2Y family and P2X7receptor in the P2X family. Available data shows that the activation of P1 receptors and P2X accelerates the OS; reversely, the activation of the P2Y family (P2Y1) causes protective effect against OS. Finally, we discuss current findings demonstrating the contribution of the purinergic signaling system to neuropsychiatric disorders and point out the potential role of OS in this process to propose a “glial purinergic-oxidative stress” (“GPOS”) hypothesis for future development of therapeutic strategies against a variety of neuropsychiatric disorders.

## 1. Introduction

After the discovery of cell energy carrier adenosine 5′-triphosphate (ATP) as a neurotransmitter, the term “purinergic” was introduced by Burnstock for the first time in 1972 [1–3]. Years later, the purinergic receptors were identified in succession. Until now, there are two families of receptors that have been identified, including P1 (adenosine activation) and P2 (ATP and its metabolite activation). P1 receptors have been classified into four groups: A1, A2A, A2B, and A3. Similarly, P2 receptors have further been categorized into P2X (1, 2, 3, 4, 5, 6, and 7) and P2Y (1, 2, 4, 6, 11, 12, 13, and 14) [4–6]. ATP is released through various mechanisms into the synaptic cleft and then hydrolyzed into ADP, AMP, and adenosine, to further bind to different receptors, triggering a series of intracellular signaling pathways that have been well investigated to establish a “purinergic signaling system” [7]. In the central nervous system (CNS), growing evidence shows that purinergic signaling plays a key role not only in physiological conditions but also in various central nervous disorders, referring to neurodegenerative disorders associated with Alzheimer's disease (AD) [8, 9], Parkinson's disease (PD) [10], Huntington's disease (HD) [11], and Amyotrophic Lateral Sclerosis (ALS) [12] and neuropsychiatric disorders, including schizophrenia (SZ) [13], major depression disorder (MDD), [14], autism [15]. Notably, one of the important underlying mechanisms associated with the regulatory effect of the purinergic system in a variety of brain disorders is the interactive effect between the purinergic system and oxidative stress (OS).

OS is a pathological condition produced by the imbalance between oxidants and antioxidants in a living system and has been recognized as one of most important pathogenetic factors for multiple brain disorders. On the one hand, repetitive exposure to oxidative stress accelerates a cascade of intracellular events, including mitochondrial dysfunction, DNA and mDNA impairment, and neuroinflammation, which, in turn, cause even more ROS production [16]. On the other hand, the oxidative stress-induced byproducts from the cell could further diffuse in the extracellular space to affect other tissues, exacerbate neuronal damage, and contribute to development of neurodegenerative and neuropsychiatric disorders [17, 18 ]. Despite the “neuron doctrine” that has governed brain research for a long time, the importance of glial cells as architects in CNS has been gradually recognized and rapidly expanded in past decades [19]. The interplay between OS and glial cells is bidirectional. OS could trigger the activation of glial cells, leading to inflammation [20]. Reversely, chronic inflammation further induces cellular OS via inflammatory cytokines [21, 22].

However, whether glial purinergic signaling interaction with the oxidative/antioxidative system participates as a major pathway in the development and therapeutic of neuropsychiatric disorders is still unclear. Therefore, the aim of this review is to firstly summarize the regulatory effect of the glial cell in response to OS and shed light on the involvement of glial purinergic signaling in this process to establish a new pathological hypothesis of “GPOS” (glial purinergic signaling-mediated oxidative stress). Then, we will discuss the potential application of this hypothesis in neuropsychiatric disorders with an emphasis on schizophrenia (SZ) and major depressive disorder (MDD).

## 2. Glial Purinergic Signaling-Mediated Oxidative Stress (“GPOS”)

The hypothesis of GPOS is mainly based on evidence shows the association of glial cells with the balance of oxidant/antioxidant and regulation of the glial-specific purinergic signaling system in this process.

### 2.1. Glial Cells and Oxidative Stress

The brain, as a high oxygen consumption organ, is particularly subjective to OS, resulting in ROS production. Overaccumulation of ROS damages DNA, lipids, and proteins, ultimately contributing to necrosis and apoptosis. Besides neurons, the crucial role of glial cell-related OS also has been comparatively investigated in the past decade. However, different types of glial cells not only share the same mechanism to produce OS but also display their unique feature to deal with OS.

#### 2.1.1. Microglia, Astrocyte, and Oligodendrocyte Share the Same Intracellular Pathway to Generate and Resist OS

Neuroinflammation and OS are common properties of neurodegenerative diseases in CNS. Neuroinflammatory stimuli can lead to elevation of reactive oxygen and nitrogen species (ROS and RNS), causing neuronal damage. Reversely, neuronal damage induced the release of proinflammatory factors which further act as a trigger to generate more OS to shape a feed-forward loop of neurodegeneration. Therefore, it is reasonable to assume an interactive pathway between inflammation and OS.

In fact, increasing evidence has confirmed that ERK/NF-*κ*B, P38 AMPK/NF-*κ*B, and JNK/BF-*κ*B exert the basic pathway to generate RNS and ROS in response to inflammatory stimuli in microglia, astrocyte, and oligodendrocyte. In microglia, activation of murine microglial cell lines (N11 and BV-2) with LPS and interferon-gamma (IFN-*γ*) caused the induction of inducible nitric oxide synthase (iNOS), subsequently increasing nitric oxide (NO) release to the surrounding environment [23]. As the two most common inflammatory signaling pathways, MAPKs and NF-*κ*B were associated with iNOS/NO induction [24, 25]. Additionally, the transfection rat microglial cell line with TAK1, the upstream kinase to activate MAPKs and BF-*Κ*B pathways and its activator protein, TAK1-binding protein 1 (TAB1), caused iNOS promoter-reporter construct activity in microglia through p38 MAPK-, JNK-, and NF-*κ*B-dependent manner [26]. Meanwhile, protein kinase C (PKC) also regulated iNOS production via MAPK and NF-*κ*B pathways in an isoform-dependent fashion in reactive microglia [27]. The generation of ROS also associated with activation of these intracellular pathways. Treating BV-2 cell lines with fluoride led to the increase in ROS partly via increasing the JNK phosphorylation level. The increasing levels of intracellular O_2_ could be markedly reduced by using the JNK inhibitor P600125 [28]. Efforts have also been made to investigate further downstream pathways. It turned out that LPS-induced mitochondrial ROS generation not only activated MAPKs, including ERK, JNK, and p38, but also regulated I*κ*B activation and NF-*κ*B nuclear localization [29]. In primary human fetal astrocyte culture, direct interleukin- (IL-) 1 stimulation increased iNOS expression via activation of NF-*κ*B [30]. The source of IL-1 could be from microglia, since the study suggested that gp41 only could trigger iNOS mRNA expression and NO production in astrocyte in the presence of microglial cell IL-1 expression [23, 31]. Zn^2+^ application also augmented LPS-induced NO production by the phosphorylation of p38 MAPK and activation of NF-*κ*B in rat astrocytes [32]. In response to LPS-induced inflammation, tyrosine kinase Fyn regulated iNOS expression via modulation of ERK phosphorylation [33]. Matrix metalloproteinase- (MMP-) 9, one of the zinc-dependent endopeptidases, has been shown to raise an impact on cell migration and inflammation modulation [34]. OS, especially the (NOX)/ROS-dependent pathway, is essential for MMP-9 expression under various stimuli. Japanese encephalitis virus-induced expression of the MMP-9 in rat brain astrocytes (RBA-1 cells) has been tested via the generation of ROS, followed by activation of p38, p42/p44 MAPK, and JNK1/2, subsequently leading to NF-*κ*B activation [35]. Furthermore, stimulating RBA-1 cells with LPS elevated MMP-9 expression and boosted the cell migration via the (NOX)/ROS-dependent NF-*κ*B pathway [36]. In agreement with microglia and astrocyte, the OS-induced MAPK pathway is also present in the oligodendrocyte. Early research found H_2_O_2_-induced oligodendrocyte death mainly through the activation of MAPK, ERK1/2, and p38 pathways [37].

Likely, these three types of glial cells also implicated the same pathway against OS, called antioxidative pathway. It is well known that activated nuclear factor erythroid 2-related factor 2 (Nrf2) could translocate and bind to the antioxidant response element (ARE), subsequently regulating the expression of a large battery of genes involved in the cellular antioxidant and anti-inflammatory defense. Meanwhile, Nrf2 is regulated by its negative regulator Kelch-like ECH-associated protein 1 (Keap1). Therefore, the Keap1/Nrf2/ARE signaling pathway was recognized as the classical antioxidative and anti-inflammatory pathway [38]. Glial cells also comply with this pathway in response to OS. In BV2 microglia, kolaviron produced antioxidant effect by increasing HO-1 via the Nrf2/ARE pathway [39]. Antioxidation and anti-inflammatory effect of icariin also depended on the activation of Nrf2 signaling in microglia [40]. In line with microglia, activation of the Nrf2/ARE pathway in astrocyte reduced OS in Parkinson's disease model [41], spinal cord injury [42], and AD [43]. In oligodendrocyte, release of Nrf2/ARE protected the oligodendrocyte from axonal damage, demyelination, and neuroinflammation [44]. Interestingly, the Nrf2/ARE pathway could partially work together with peroxisome proliferator-activated receptor gamma (PPAR-*γ*) to preserve mitochondrial function, defend against OS, and promoted OPC differentiation [45]. PPAR-*γ* self-activation induced a protective effect on oligodendrocyte mitochondria attributed to the elevation of the expression of PGC-1*α* (a mitochondrial biogenesis master regulator), UCP2 (a mitochondrial protein known to reduce ROS production), and cytochrome oxidase subunit COX1 and influenced oscillatory Ca^2+^ waves to defend against TNF-*α* damage [46, 47].

#### 2.1.2. Microglia, Astrocyte, and Oligodendrocyte Displayed Their Unique Features in Response to OS

NADPH oxidases (NOXes) are one of the major sources of cellular ROS. Under physiological condition, suitable regulation of NADPH oxidase activity is crucial to maintain a healthy level of ROS in the body. However, overactivity of these enzymes could overproduce ROS, which further leads to OS and cell damage. To date, there are 7 human isoforms of the complex, including NOX1, NOX2, NOX3, NOX4, NOX5, DUOX1, and DUOX2. Interestingly, different types of glial cells showed different expression patterns which could implicate distinct therapeutic potential.

The experiments performed on neuron-glia mixed culture from NADPH oxidase-deficient (PHOX-/-) and wild-type mice demonstrated that LPS-induced dopaminergic neurotoxicity mainly was caused by microglial activation through NADPH oxidase, producing the ROS accumulation [48]. Among the NOX family (NOX1–5 and DUOX1–2) [49], NOX 2 was highly expressed in microglia. In contrast, expression of NOX1 and NOX4 in microglia is still controversial due to the lack of specific antibodies [50]. Although evidence showed the increase in both NOX2 and NOX4 levels in microglia-neuron mixed culture exposed to iron and LPS [51], the spotlight has been focused on NOX2. By employing p47^phox^-deficient mice, a study demonstrated that the functional subunit of NOX2 switched microglia to an activation state in response to an inflammatory challenge [52]. With an exception of p47^phox^, the increased expression of the p67^phox^ subunit also has been observed when N9 microglia lines and primary microglia culture were treated with angiotensin II [53]. Unlike microglia, the expression of NOXs in astrocytes and oligodendrocytes is quite low. In astrocyte, NOX is activated by PKC and intracellular calcium. The activity of NOX could be modulated by intracellular pH environment, suppressed by intracellular alkalinization, and enhanced by acidification [54]. Regardless of less expression of NOXs, treatment of oligodendrocytes with injured astrocyte media with a reduction in zinc levels was sufficient to result in NADPH oxidase activation by gp91 phox [55]. A study by using the cell line MO3-13 displaying the molecular and cellular features of OL precursors was performed to determine the effect of NOXs on oligodendrocyte differentiation. The elevated expression of NOX3 and NOX 5 in human OLs was observed for the first time after H_2_O_2_ treatment, and selective depletion of these proteins inhibited differentiation induced by the protein kinase C (PKC) activator, phorbol-12-myristate-13-acetate (PMA). Furthermore, NOX5 silencing down-regulated NOX3 mRNA levels, suggesting that ROS produced by NOX5 upregulated NOX3 expression [56].

Another distinct feature for different types of glial cells is their antioxidant gene expression, especially the expression of glutathione (GSH), a major cellular antioxidant and redox regulator in the brain against OS. Microglia and astrocyte showed higher expression compared to oligodendrocyte [57, 58]. This feature makes oligodendrocyte more susceptible to OS in comparison with microglia and astrocyte. Another enzyme, which could inhibit ROS production, is SHP-1, a non-receptor-type protein tyrosine phosphatase. The presence of SHP-1 had an inhibitory effect on the activation of transcription factors NF-*κ*B, STAT3, and STAT6, further suppressing ROS production [59, 60]. The expression of SHP-1 mainly depended on the physiopathological conditions. Although the expression of SHP1 could dramatically increase in all types of glial cells under pathological stimulation, the physiological expression of SHP1 varied from microglia and astrocyte to oligodendrocyte. Under physiological condition, immunohistochemistry showed that SHP1 immunoreactivity colocalized with GFAP-positive astrocytes, but not with microglia [61]. In either mixed glial culture or pure culture, oligodendrocytes expressed high levels of SHP-1 in the cytoplasm of cell bodies and processes [62]. The unique expression pattern probably could implicate a dominant antioxidative effect of astrocyte and oligodendrocyte compared to microglia.

### 2.2. Glia Purinergic Signaling-Mediated OS

#### 2.2.1. P1 Family Member Adenosine and Its Receptors Exerted an Opposite Effect in Response to OS

Under physiological condition, extracellular ATP could be rapidly converted into ADP, AMP, and adenosine in the presence of enzymes CD39 and CD73. To keep the homeostasis, the nucleoside transporters (ENTs) expressed on the membrane of glial cells take up the excessive adenosine from extracellular into intracellular part. Intracellular adenosine is either further hydrolyzed to inosine by adenosine deaminase or phosphorylated to AMP by adenosine kinase (ADK) to efficiently regulate adenosine levels. In an OS-loaded brain, the elevated extracellular adenosine could significantly protect neurons from damage. The study from cultured astrocytes found that OS induced extracellular adenosine accumulation partially via decreasing the function of ENT1 without expression alteration [63]. However, pretreating microglia with adenosine could significantly decrease H_2_O_2_-induced ROS overproduction compared to the nontreated group. This benefit is accompanied by expression of antioxidative enzyme hemeoxygenase-1 (HO-1) expression though Nrf2/ARE and PI3K/Akt pathways. Furthermore, the effects of adenosine are independent of its receptors, since pharmacological activation and inhibition of A1, A2A, and A3 had small impact on ROS generation and HO-1 expression [64].

Inconsistent with the beneficial effect of adenosine itself, it exerts a harmful effect on OS once it activates its receptors. For example, extracellular adenosine activated A3 receptors, which further caused OS and disturbed mitochondrial membrane potentials in oligodendrocytes [65]. After retinal detachment, blockade of A2A receptors could protect the photoreceptor by inhibiting microglia proliferation, decreasing IL-1, and suppressing ROS overproduction [66]. In LPS-treated mixed glia cultures with astrocyte and microglia, the A2A agonist CGS21680 potentiated LPS-induced NO release and NO synthase II expression, and the potentiation was inhibited by the A2A antagonist ZM-241385 [67].

#### 2.2.2. P2X Family Especially P2X7 Receptor Activation Exacerbated OS

The P2 receptor family also participates in the regulation of OS. Among the P2X subgroup, most of interests have been focused on P2X7 receptors due to their unique low affinity to ATP, especially under pathological conditions. Using mixed neuron and microglia culture from wild-type and P2X7-deficient mice confirmed that microglial P2X7 receptor activation dramatically increased the release of superoxide and nitric oxide, contributing to cortical neuronal death [68]. Further experiments from *β*-amyloid-stimulated microglia indicated that microglial P2X7 activation could not only induce the release of superoxide but also increase the level of ROS. This experiment uncovered the important contribution of the influx of extracellular Ca^2+^ to the production of ROS. Interestingly, the source of Ca^2+^ influx was attributed to P2X7 activation induced by *β*-amyloid-stimulated ATP release directly from microglia in an autocrine fashion [69]. However, P2X7 activation-induced ROS production also could be Ca^2+^ independent. In the murine microglial EOC13 cell line, researchers found that cells had higher ATP-induced ROS formation in the absence of Ca^2+^ compared to the presence of Ca^2+^ without P2X7 function alteration. Results were confirmed by incubation of the Ca^2+^ chelator EGTA. Similarly, high extracellular K^+^ incubation also was unable to impair P2X7-induced ROS formation [70]. P2X7-induced ROS production could be influenced by extracellular acidification in different ways in the BV-2 microglial line. Short-time acidification efficiently suppressed maximal ionic current response of P2X7 at the upstream level. However, long-lasting acidification induced intracellular OS by coordinating enhancement with P2X7 activation for mitochondrial toxicity [71].

The intracellular pathways of P2X7 activation-induced OS also have been investigated. In primary rat microglia culture, both ATP and P2X7 agonist BzATP stimulations caused the production of superoxide by activation of NADPH oxidase and pharmacological inhibition of P38 MAPK attenuated the superoxide production. This experiment indicated that microglial P2X7 activation induced cortical neuron death by the NADPH-P38 MAPK pathway [72]. Although the ATP/P2X7/ROS-/ASK1/p38 pathway has been identified in macrophage [73], other studies addressed this pathway in microglia, the resident immune cells of the CNS. Therefore, BV2 and MG6 microglia lines were used for this purpose. By using the immunoblot method, researchers found ATP-induced P38 phosphorylation which could be reversed by the p38 inhibitor SB, ROS scavengers, and P2X7 inhibitor CBB. Together with all the results, it turned out that P2X7 activation-induced ROS generation was a necessary process to activate the ASK1-p38 pathway. Furthermore, the CaMKII inhibitor KN-93 that suppressed ASK1 activation, p38 activation, and cell death further identified the upstream signaling of ASK1-p38 [74]. Compared to the MAPK signaling pathway which was largely related to cellular proliferation, differentiation, and survival, the AMPK signaling regulates cellular metabolism and cellular energy homeostasis by monitoring the AMP : ADP : ATP ratio [75]. Therefore, to uncover if P2X7 activation-induced OS via the AMPK pathway further contributes to mitochondrial dysfunction will be particularly important in future. The influx of Ca^2+^ and efflux of K^+^ are two major upstream events after P2X7 receptor activation. Intracellular overload of Ca^2+^ together with robust efflux of K^+^ interrupted the cytosol pH and mitochondrial electron transport, followed by ROS overproduction. To our knowledge, ROS overproduction parallel with Ca^2+^/CaMKII resulted in phosphorylation of AMPK. The consequence of AMPK activation further increased mitochondrial fission and caused cell death in cultured BV-2 microglia cells [76]. In astrocyte, P2X7 receptor activation increased ROS production through NADPH oxidase, subsequently leading to IL-6 release [77].

#### 2.2.3. P2Y Family Especially P2Y1 Receptor Activation Alleviated OS

Parallel with ion channel P2X receptors, the G protein-coupled P2Y family is also associated with regulation of OS in a different way. In cultured astrocytes, short-time ATP incubation displayed a protective effect against H_2_O_2_-induced cell death. However, preapplication of the P2Y1 receptor antagonist MRS2179 efficiently reversed the protective action, which confirmed the involvement of P2Y1. The results from DNA microarray analysis and quantitative RT-PCR analysis demonstrated that ATP incubation upregulated oxidoreductase genes such as TrxR, CBR, and superoxide dismutase-like gene. However, detailed connection between P2Y1-related protection and the upregulated genes was still unclear [78]. In coculture with astrocyte and neurons, they found that the protective effect induced by P2Y1 receptor activation against H_2_O_2_ attributed to IL-6 release from astrocyte instead of neuron [79]. The mechanism of P2Y1 induced protective effect against H_2_O_2_ in astrocyte has been tested. Researchers found that H_2_O_2_ evoked the activation of src tyrosine kinase, which further enhanced ERK1/2 phosphorylation, resulting in cell death. Therefore, inhibition the activation of src tyrosine kinase was a crucial process to protect the cell from death. Remarkably, P2Y1 agonist 2MeSADP enhanced the gene expression and activity of protein tyrosine phosphatase (PTP), which was responsible for the inhibition of src tyrosine kinase. Thus, P2Y1-PTP- src tyrosine kinase-ERK1/2 pathway could be essential for the protective effect [80]. In addition, there are still some other mechanisms showing the involvement of P2Y1 in OS. In mixed hippocampal culture with astrocyte and neurons, P2Y1 activation directly contributed to ROS decrease and mitochondrial depolarization [81]. With an exception of P2Y1 receptor, other P2Y receptors in astrocytes also have been showed to regulate the OS. The P2Y12/13 agonist 2MeADP application decreased ROS generation and mitochondrial depolarization. In contrast, the P2Y6 agonist UDP increased ROS production, whereas that of P2Y2/4 did not show any effect on ROS overproduction [82]. In astrocyte-microglia interaction experiment, UDP activated microglia P2Y6 and further coupled to the phospholipase C (PLC)/PKC pathway, which mediated an increase in inducible nitric oxide synthase (iNOS) expression and in nitric oxide (NO) release. Consequently, the diffusible NO mediated astroglial apoptosis [83].

## 3. GPOS in Neuropsychiatric Disorders

### 3.1. GPOS in SZ

Schizophrenia (SZ) is a common psychiatric disorder characterized by positive, negative, and cognitive symptoms. It has been identified that genetic susceptibility factors combined with environment insults together contribute to the development of SZ. To date, several pathogenesis models of SZ have been proposed from different aspects. (1) Cell-specific involvement, including hyperdopaminergic, hypoglutamatergic, hypo-GABAergic, and hyposerotoninergic; (2) gene-related involvement; (3) energy metabolism disturbance [84, 85]; and (4) oxidative and antioxidant imbalance [86].

Based on evidence suggesting that purine derivative allopurinol administration exerted a beneficial therapeutic effect on patients with SZ, Lara and colleagues first proposed a purinergic hypothesis of SZ [87, 88]. To date, the participant of the purinergic system in SZ is mainly related to adenosine receptors (P1 family), P2X7 receptors (P2X family), and P2Y1 receptors (P2Y family). Purinergic receptor activation or inhibition affected the behavior alteration in SZ. A clinical study supported that the reduction of A2AR levels accompanied by an altered motor phenotype in a subgroup of SZ patients, but A1 kept unchanged [89]. Few clinical data are available regarding P2Y and P2X receptors participation in SZ pathophysiology. By using bilateral microinfusions of the selective agonist MRS2365 into the medial prefrontal cortex (mPFC), the study addressed that activation of P2Y1 in mPFC reduced prepulse inhibition (PPI) while having no impact on startle amplitude [90]. Two antipsychotics drugs, prochlorperazine and trifluoperazine, acted as a negative allosteric modulator to inhibit human P2X7 receptor function [13]. Furthermore, in the phencyclidine-induced SZ model, genetic deletion and pharmacological inhibition of P2X7Rs alleviated schizophrenia-like behavioral alterations, increasing social interactions and alleviating hyperlocomotion and stereotype behavior [91, 92].

However, the underlying mechanism mainly focused on purinergic receptor polymorphisms and interaction with dopamine receptors and NMDA receptors. The link between adenosine A2A receptor gene polymorphism located on chromosome 22q and susceptibility to SZ was reported [93, 94]. By measuring the polymorphisms in ADORA1, ADORA2A, and ADORA3, a study found a correlation between ADORA1 rs3766566 and positive psychopathological symptoms, ADORA2A rs2298383 and general psychopathological symptoms, and ADORA2A rs5751876 and akathisia [95]. In contrast, no association between SZ and polymorphisms of P2X7 receptor has been observed so far [96].

The dopamine hypothesis of SZ is largely based on the effects of D2R antagonists and agonists to alleviate and to accelerate the symptoms, respectively. The interaction between A2A and dopamine D2 [97] and A2A and NMDA [98] made A2A a potential target to SZ. By forming A2A/D2 heteromers, the reduction of adenosine level caused the elevation of dopamine level in SZ [99, 100]. Besides adenosine receptors, P2Y (P2Y1) and P2X (P2X7) also have been found to associate with SZ mainly through interacting with dopamine receptors and glutamate receptors. The emerging experimental evidence found that stimulation of P2Y1 receptors was related to elevation of dopamine release [101]. In contrast, activation of these receptors also caused the hypofunction of NMDA [102]. Importantly, the activation of dopamine receptor D2 could diminish the excessive production of ROS and RNS induced by bradykinin, a proinflammatory B2R-activating peptide. Together with the previous evidence implicating that A2A activation accumulated ROS, it suggested that A2A/D2 heteromers may play a complex role in the regulation of OS in SZ. In animal study, A1 receptors agonists could protect against neuropathological changes in rat retrosplenial cortex after administration of the NMDA receptor antagonist MK-801 [103]. It has been reported that MK-801 administration caused ROS and RNS production in rat [104, 105]. However, whether A1-induced protective effect involves the regulation of ROS and RNS is still masked due to the lack of direct evidence.

It is worth noting that glial expressed purinergic receptors probably devote to the pathogenesis of SZ. In the study of mice with A2A receptors deficiency from astrocytes, MK-801-induced inhibition of psychomotor functions and memory as well as suppression of glutamine transporter activity was observed [106]. This study revealed the expression of A2A receptors in astrocyte contributed to MK-801 induced psychotic symptoms. Under normal condition, the expression of A2A in astrocyte is lower than in certain population of neurons. The A2A expression could dramatically increase when responded to pathological stimulation. For example, study found that astrocytic A2A but not microglial A2A is increased in AD model [107]. Compared to the expression and function of microglia P2Y1, the role of astrocytic P2Y1 is less debated as it turned out to perform a variety of brain functions by regulating neuron-to-glia communications. With regard to P2X7, the research spotlight has been put on glial cells since the function or existence of neuronal P2X7 receptor remained a matter of debate [108, 109]. However, recently it was reported that there is no detectable microglia activation and neuroinflammation under subchronic PCP treatment [91]. Nevertheless, this study did not check the activation of glial cells in a chronic model and OS markers. In a rat SZ model, PCP administration could generate OS in hippocampus by measuring OS marker nitrotyrosine and chronic antipsychotics quetiapine application could significantly attenuate OS and object recognition memory impairment [110]. Therefore, more direct evidence should be provided in future to elucidate whether the regulation of P2X7 on PCP-induced SZ via glial involved OS.

### 3.2. GPOS in MDD

Major depressive disorder (MDD) is a common psychiatric disease prevalent worldwide and characterized by severe and persistent emotional symptoms (feelings of guilt and anhedonia), cognitive symptoms (low self-esteem) and somatic symptoms (loss of sleeplessness and mental irritancy). Compared to SZ, purinergic signaling also received relatively considerable attention in MDD. The excellent review from Bartoli et al. elaborately summarized the promising role of the purinergic system in depression, highlighting potential antidepressant effect of A2A and P2X7 selective antagonists and detection of purinergic system peripheral metabolites as biomarkers of depression [111].

Recent reviews have also summarized the evidence from both human and animal studies concerning the involvement of the adenosinergic system in pathophysiology and treatment of MDD [112, 113]. In these two reviews, authors collected evidence in support of the involvement of the adenosinergic system in MDD from adenosine synthesis, adenosine cleanup, and catabolism to adenosine receptor regulation. Herein, we only focused on the glial-related purinergic system in MDD. It is well known that sleeplessness, one of typical symptoms of MDD, was related to the level of adenosine [114, 115]. Sleep deprivation for 12 h led to the elevation of adenosine levels in the rodent frontal cortex. Importantly, astrocyte was capable of modulating changes in nonrapid eye movement slow wave activity in response to sleep deprivation [116, 117]. Another study found that selective expression of dnSNARE (dominant negative SNARE domain of the vesicle protein VAMP2) in astrocytes reduced extracellular adenosine accumulation mediated by the A1 receptor [118]. Wakefulness is related to high neuronal metabolism to maintain neuronal activity, which requires a great amount of oxygen, further resulting in oxidant production. Thus, sleep was a particularly important process for the brain to recover or increase antioxidant activity against free radicals such as ROS and RNS [119]. Together, we speculate that the regulation of the astrocytic A1 receptor in sleep might undergo OS pathways.

Along with adenosine, ATP signaling via the P2 receptor might also play a pivotal role in the neuropathological mechanisms of MDD. The blockade of P2 receptors has been shown antidepressant-like effects in the animal model [120]. Among P2 receptors, the P2X7 receptor has received lot of research interest. Polymorphism research pointed out the association of nonsynonymous coding of single-nucleotide polymorphism (SNP) rs2230912 in the P2X7 gene with MDD [121, 122]. Evidence showed an antidepressant-like profile and higher responsivity to the antidepressant treatment in P2X7 knockout mice [123, 124]. Most of studies emphasized that the antidepressant effect induced by P2X7 blockage was a result of monoamine and glutamate regulation [120, 121]. Indeed, P2X7 activation induced neuroinflammation might contribute to pathogenesis of MDD, although the role of direct neuronal mechanisms cannot be excluded either.

P2X7 receptor and its mediated signaling pathway play an important role in microglia activation. In microglia, there are two major inflammatory pathways involved into P2X7 activation, including P2X7/NLRP3/IL-1*β* [125] and P2X7/NF-*κ*B/IL-1*β*. Chronic uncontrolled stress was a major cause of depression, which leaded to the activation of caspase-1 by the NLRP3 inflammasome, followed by production of inflammatory cytokine such as IL-18 and IL-1*β* [126]. In MDD model, the increased expression of NLRP3 inflammasome mRNA and IL-1*β* have been detected after LPS application, suggesting NLRP3 inflammasome and IL-1*β* acted as mediators of inflammation [127]. Similarly, the enhancement of IL-1*β* signaling in the hippocampus leaded to the development of depressive symptoms [128]. P2X7 activation could directly activate NLRP3, further resulting in IL-1*β* release. Besides this, the activation of inflammasome in microglia required potassium efflux, ROS production and cathepsin B function [129]. In particular, inflammasome-activating signal reactive oxygen species (mtROS) served as direct activator of the NLRP3: ASC: pro-Caspase-1 complex [130]. Another essential element for NLRP3-inflammasome activation is the transcription factor NF-*κ*B, which acts downstream of TLRs and other immune receptors. Surprisingly, P2X7 activation also served as the upstream signaling molecule of NF-*κ*B [131]. NF-*κ*B signaling could be activated and repressed by ROS in a phase and context dependent manner. The NF-*κ*B pathway can have both anti- and prooxidant roles in the setting of OS [132]. Together with previous evidence showing that P2X7 activation caused ROS overproduction, we could assume that P2X7 activation induced inflammation pathway probably interacts with the OS pathway, contributing to the development of MDD.

## 4. Conclusive Remarks and Perspective

**Figure 1:**
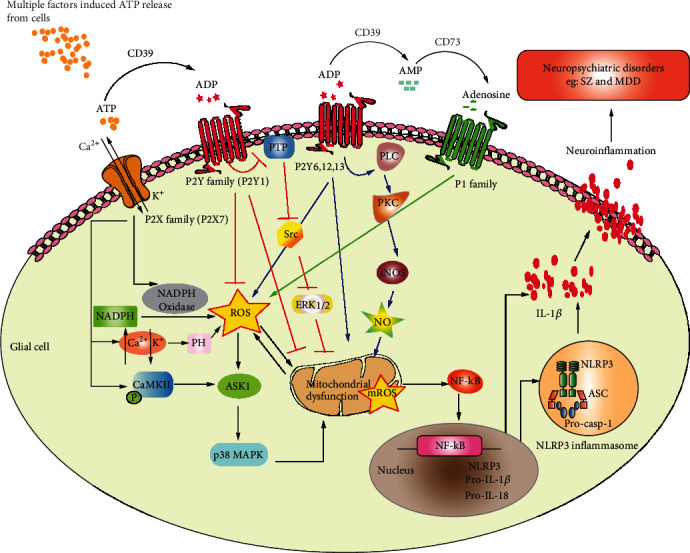
“Glial purinergic-oxidative stress” (“GPOS”) hypothesis in neuropsychiatric disorders (SZ and MDD). Multiple factor-induced ATP release from cells, on the one hand, is hydrolyzed into ADP, AMP, and adenosine in the presence of CD39 and CD73. On the other hand, ATP, ADP, and adenosine receptors triggering an intracellular pathway. The P2X family especially P2X7 activation by ATP increases the ROS through several pathways, including to activate NADPH oxidase, to adjust pH level, and to phosphorylate CaMKII. The increase in ROS further activates ASK1/p38 MAPK, subsequently resulting in mitochondrial dysfunction. In the P2Y family, P2Y1 exerts a protective role in suppressing ROS overproduction through inhibiting the PTP/Src ERK1/2 pathway. In contrast, P2Y6, 12, and 13 increase ROS by activating PLC/PKC/NO. P1 family activation also could elevate the ROS production. ROS overproduction interacts with mitochondrial dysfunction; could activate and translocate BF-*κ*B to the nucleus and increase inflammatory cytokine gene expression, especially IL-1*β*, IL-8, and NLRP3. After NLRP3 inflammasome assembly, mature IL-1*β* and IL-8 could be released from the glia cell and cause neuroinflammation further contributing to neuropsychiatric disorders such as SZ and MDD.

Based on evidence showing that the purinergic system participates in the regulation of oxidative/antioxidative pathways in glial cells, we proposed a new neuropathological hypothesis termed as “GPOS” (glial purinergic system-mediated OS) for the first time. Then, we collectively elucidated the participation of the glial purinergic system in SZ and MDD and discussed the potential application of “GPOS” in neuropsychiatric disorders (Figure 1). However, there are still questions to be answered in support of this hypothesis. First of all, the direct evidence should be provided to confirm the role of “GPOS” in neuropsychiatric disorders and in other neurodegenerative disorders. Secondly, cell type-specific or subgroup dominant regulation of the purinergic system should be addressed by using multiple genetic manipulation approaches. Thirdly, given the correlation between redox status and the vitagene network and its possible biological relevance in neuroprotection, it is important to further address whether it is a hormesis-dependent mechanism in neurodegenerative/neuroprotective disorders [133–136]. Whether the purinergic system alters oxidant/antioxidant balance or hormesis response in a neurodevelopment manner also should be further confirmed. And whether or not GPOS will be a potential promising target for the treatment of SZ and MDD, it is necessary to shorten the gap between the basic and translational research.
